# Urban residential building stock synthetic datasets for building energy performance analysis

**DOI:** 10.1016/j.dib.2024.110241

**Published:** 2024-02-28

**Authors:** Usman Ali, Sobia Bano, Mohammad Haris Shamsi, Divyanshu Sood, Cathal Hoare, Wangda Zuo, Neil Hewitt, James O'Donnell

**Affiliations:** a School of Mechanical and Materials Engineering and UCD Energy Institute, UCD, Dublin, Ireland; b School of Architecture and The Built Environment, Ulster University, Belfast, UK; c Pennsylvania State University, University Park, PA, USA; d Flemish Institute for Technological Research (VITO), Boeretang Mol, Belgium

**Keywords:** Building energy performance, Urban building energy modeling, Building retrofit, Building features

## Abstract

The urban building stock dataset consists of synthetic input and output data for the energy simulation of one million buildings. The dataset consists of four different residential types, namely: terraced, detached, semi-detached, and bungalow. Constructing this buildings dataset requires conversion, categorization, extraction, and analytical processes. The dataset (in .csv) format comprises 19 input parameters, including advanced features such as HVAC system parameters, building fabric (walls, roofs, floors, door, and windows) U-values, and renewable system parameters. The primary output parameter in the dataset is Energy Use Intensity (EUI in kWh/(m^2^*year)), along with Energy Performance Certificate (EPC) labels categorized on an A to G rating scale. Additionally, the dataset contains end-use demand output parameters for heating and lighting, which are crucial output parameters. jEPlus, a parametric tool, is coupled with EnergyPlus and DesignBuilder templates to facilitate physics-based parametric simulations for generating the dataset. The dataset can be a valuable resource for researchers, practitioners, and policymakers seeking to enhance sustainability and efficiency in urban building environments. Furthermore, dataset holds immense potential for future research in the field of building energy analysis and modeling.

Specifications Table

**Table:** 

Subject	Engineering
Specific subject area	Urban Building Energy Modeling (UBEM)
Data format	CSV format
Type of data	Table
Data collection	The dataset was generated synthetically through physics-based parametric simulations using jEPlus, a parametric tool that is coupled with EnergyPlus and DesignBuilder templates. The data were collected for various residential building archetypes, including terraced houses, detached houses, semi-detached houses, and bungalows.
Data source location	University College Dublin, Ireland
Data accessibility	Repository name: Urban Building Energy Stock Datasets (Mendeley Data) Data identification number: 10.17632/m6vv9k9gcd.1 Direct URL to data: https://data.mendeley.com/datasets/m6vv9k9gcd/
Related research article	Usman Ali, Sobia Bano, Mohammad Haris Shamsi, Divyanshu Sood, Cathal Hoare, Wangda Zuo, Neil Hewitt, James O'Donnell. “Urban building energy performance prediction and retrofit analysis using data-driven machine learning approach”. Energy and Buildings, Volume 303, https://doi.org/10.1016/j.enbuild.2023.113768

## 1 Value of the Data

• The urban residential building stock synthetic dataset is valuable to the scientific community as it provides a comprehensive and detailed set of data for analyzing the energy performance of 1 million urban buildings. Researchers can use this dataset to gain insights into energy consumption patterns and efficiency in urban residential structures.
• The dataset includes four different types of Irish residential buildings, mainly focusing on Dublin City, allowing researchers to study a wide range of building characteristics and energy profiles. This diversity enhances its value for various research applications. This highlights the novelty and local specificity of the dataset.
• The dataset generated for the Irish building stock, mainly focusing on Dublin City, was developed before establishing a regional database covering all dynamic building parameters. This highlights the novelty and local specificity of the dataset.
• The datasets contain 19 input parameters, including detailed information on HVAC systems, building fabric properties (U-values for walls, roofs, floors, doors, and windows), and renewable energy systems, these data enable researchers to perform sophisticated energy performance analyses and simulations.
• The primary output parameter, EUI (kWh/(m^2^*year)), provides a crucial metric for evaluating and comparing the energy efficiency of different buildings. This information can inform energy-efficient building design and retrofitting efforts.
• The inclusion of EPC data, categorized on an A to G rating scale, allows researchers to assess the overall energy performance and certification status of the buildings in the dataset The dataset also contains output parameters for heating and lighting, which are essential for understanding specific energy end-uses within residential buildings. This information can guide energy-saving strategies and policy development. These data have the potential to support sustainability research and can be used to evaluate and develop strategies for enhancing sustainability and efficiency in the urban built environments. Policymakers and practitioners can leverage this dataset to inform urban planning and energy management initiatives.

## 2 Background

Stakeholders play a crucial role in analyzing the energy performance of urban buildings to develop effective policies for mitigating energy consumption and reducing CO_2_ emissions. However, the task of collecting and analyzing energy data for buildings on a large urban scale is complicated and time-consuming, demanding substantial resources. To address this challenge, we employ a methodology to generate synthetic urban building stock data through a data-driven and parametric simulation approach. These datasets are then utilized in implementing a data-driven machine learning strategy to predict the energy performance of urban residential buildings, encompassing both ensemble-based machine learning and end-use demand segregation methods. The datasets contain the relevant parameters, including heating, lighting, equipment, photovoltaic, and hot water, providing valuable support to stakeholders such as energy policymakers and urban planners in making well-informed decisions for large-scale retrofitting initiatives.

## 3 Data Description

This dataset, comprising over 1 million rows and 32 columns, offers a detailed analysis of residential urban building stock, focusing on energy efficiency and building characteristics. It serves as a helpful resource for understanding urban residential buildings, particularly in the context of energy consumption and efficiency (Table 1). The dataset contains 19 building input features (Table 2). Half of the dataset is simulated based on the 2030 Dublin weather file data, while the other half is based on historical Dublin weather file data, providing a comprehensive statistical climate analysis. The dataset includes Irish building stock comprising Bungalows (27%), Detached houses (24%), Semi-Detached houses (25%), and Terraced houses (24%). This combination allows for comparative analysis across different residential structures. Approximately 60% of the buildings employ renewable energy sources, highlighting a significant shift towards sustainable energy practices.

**Table 1:** Brief Summary of urban building stock datasets for building energy performance analysis.

Category	Details	Summary
Total Records	1 million	Comprehensive dataset with a large sample size.
Total Columns	32	Divided into 19 input features and 13 output features.
Building Types	Bungalow, Detached, Semi-Detached, Terraced	Balanced representation: Bungalow (27%), Detached (24%), Semi-Detached (25%), Terraced (24%).
Weather Data	Historical,2030	Equal distribution between historical and 2030 profile.
Renewable Energy Usage	Yes, No	Significant use of renewables (60% of buildings).
Building Energy Rating	A to G, 15 categories	Most common rating: 'C' (approx. 19%).
Insulation U-Values	Floors, Doors, Roofs, Windows, Walls	Critical for assessing thermal efficiency.
Energy Efficiency Metrics	HVAC Efficiency, Building Orientation, Lighting and Equipment Density, etc.	Essential for understanding energy consumption patterns.
Energy Use Intensity (EUI)	Average 241 kWh/m²	Standardized measure for energy efficiency comparison.
Energy Consumption	Heating, Water Systems, Lighting, Equipment, etc.	Detailed insights into various aspects of energy usage.
Photovoltaic Power	Average 518 kWh	Indicates moderate solar energy utilization.
Conversion Factors	Heating (avg. 0.65), Electricity (avg. 2.08)	Efficiency of energy conversion processes.

**Table 2:** Statistical summary of all building input features across urban building stock datasets.

Feature	Units	Mean	Std Dev	Min	Max
Floor_Insulation_U-Value	W/m²K	0.37	0.28	0.15	1.6
Door_Insulation_U-Value	W/m²K	2.28	1.41	0.81	5.7
Roof_Insulation_U-Value	W/m²K	0.88	0.73	0.07	2.28
Window_Insulation_U-Value	W/m²K	2.44	1.58	0.73	5.75
Wall_Insulation_U-Value	W/m²K	1.03	0.76	0.1	2.4
HVAC_Efficiency	%	2.84	1.32	0.3	4.5
Domestic_Hot_Water_Usage	Liter/m²/day	1.65	1.15	0.5	3.5
Building_Orientation	Degree	124.8	111.85	0	315
Lighting_Density	W/ m²	4.58	2.75	1	9
Occupancy_Level	Person	3.52	1.71	1	6
Equipment_Density	W/m²	9.92	6.86	1	21
Heating_Setpoint_Temperature	°C	20.14	1.67	18	23
Heating_Setback_Temperature	°C	11.79	1.37	10	14
Air_Change_Rate	Air changes per hour	1.53	1.57	0.35	3
Window_to_Wall_Ratio	%	37.5	25.86	0	70

The dataset contains detailed information on the U-Value for floors, doors, roofs, windows, and walls. These five key metrics related to building insulation are measured in W/m²K. The average U-Value for floor insulation is 0.37, with a range from 0.15 to 1.6, indicating variability in floor insulation efficiency. Door insulation has a higher average U-Value of 2.28, with a wider range extending up to 5.7, suggesting greater diversity in door insulation quality. Roof, window, and wall insulations have average U-Values of 0.88, 2.44, and 1.03 respectively, each with a significant spread in values. Lighting density and occupancy levels are measured, averaging 4.58 W/m² and 3.52 persons respectively. Equipment density, another significant factor in energy consumption, has an average of 9.92 W/m². The dataset includes heating setpoint and setback temperatures, averaging 20.14 °C and 11.79 °C respectively, which are important for heating energy calculations. Air change rate, a measure of ventilation, averages 1.53 air changes per hour, a critical component in assessing indoor air quality and energy loss. These factors are crucial for understanding the end-use energy demands of buildings. In summary, this dataset provides a comprehensive view of the factors influencing energy performance in urban residential buildings. The range of data from insulation values to internal building factors offers a holistic understanding of the energy dynamics in residential environments. This information is invaluable for energy performance analysis and aids in identifying areas for improvement and sustainable development in urban residential architecture.

On the other hand, there are 13 output features (Table 3). The dataset outlines detailed energy consumption metrics, such as heating usage, water systems energy, interior lighting and equipment energy, and total heating energy. These parameters are vital for assessing the energy efficiency of residential buildings. This data is essential for identifying key areas of energy consumption in residential buildings. The inclusion of photovoltaic power data (averaging 518 kWh/yr) suggests a focus on solar energy utilization in these buildings. The negative values of total electricity energy indicate that the building generates energy from photovoltaics and exports it to the grid. The Heating and Electricity Primary Conversion Factors (averaging 0.65 and 2.08 respectively) offer insights into the effectiveness of energy conversion processes in these buildings. One of the most important output features is the EUI. With an average of 241 kWh/m², the EUI data helps benchmark the energy efficiency of buildings, providing a standardized measure for comparison. Overall, the Building Energy Rating spans 15 categories (A1 to G) based on the Irish Building rating standard, with 'G' being the most frequent. This suggests a wide range of energy efficiencies in urban residential buildings. All input and output features are non-null, indicating a dataset with complete information for every entry.

**Table 3:** Statistical summary of all building output features across urban building stock datasets.

Feature	Units	Mean	Std Dev	Min	Max
Total_Building_Area	m^2^	103.51	17.37	85.91	130.81
Heating_Usage	kWh/year	18,390.56	13,531.47	206.51	10,7361.3
Water_Systems_Energy	kWh/year	5956.09	7825.23	202.8	25,403.65
Interior_Lighting_Energy	kWh/year	1146.38	723.98	207.96	2879.78
Interior_Equipment_Energy	kWh/year	3066.85	2209.87	243.97	8174.23
Total_Heating_Energy	kWh/year	12,434.47	12,828.89	0	10,6752.9
Photovoltaic_Power	kWh/year	518.36	471.71	0	1233.25
Total_Electricity_Energy	kWh/year	3720.79	2549.69	-690.26	11,054.02
Heating_Conversion_Factor	Numeric	0.65	0.68	0.24	3.61
Electricity_Primary_Conversion_Factor	Numeric	2.08	0	2.08	2.08
Heating_Primary_Conversion_Factor	Numeric	1.87	0.4	1.1	2.08
Energy_Use_Intensity	kWh/(m^2^*year)	241.38	148	−9.43	650

This dataset is a valuable resource for researchers and policymakers focusing on urban residential energy efficiency. Its comprehensive nature, covering various building types and a wide range of energy-related features, makes it uniquely suited for in-depth analyses. Including historical and 2030 weather data enhances its applicability for longitudinal studies. The detailed data on building insulation, orientation, and density factors, combined with extensive energy consumption metrics, allows for a holistic understanding of energy dynamics in residential buildings. The energy ratings provide a straightforward way to gauge the efficiency of buildings, potentially guiding efforts towards energy optimization and sustainable development in urban residential sectors. Overall, the dataset not only reflects current energy practices but also serves as a guidepost for future energy sustainability initiatives in urban residential system modeling.

**Fig. 1:**
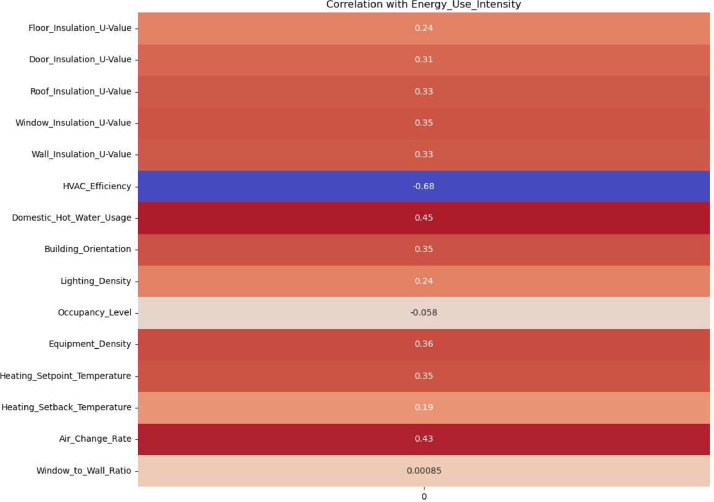
The correlation with all input parameters with final output energy use intensity.

The correlation coefficients between various input building parameters and the final output EUI in the datasets provide insights into how different factors influence energy consumption (Fig. 1). Firstly, the insulation values (U-Values) for the floor, door, roof, window, and wall show positive correlations ranging from 0.24 to 0.35. This suggests that higher U-Values, which indicate poorer insulation quality, are associated with increased EUI. HVAC Efficiency shows a strong negative correlation (−0.68) with EUI. This is the most significant correlation in the dataset and implies that higher HVAC efficiency substantially reduces energy use, highlighting the importance of efficient heating and cooling systems in residential buildings. Domestic Hot Water Usage and Air Change Rate indicate a moderately strong relationship with EUI, underscoring the energy impact of hot water systems in residential settings. Building Orientation, Equipment Density, and Heating Setpoint Temperature, both with correlations of 0.35, suggest a notable impact on EUI. In contrast, Occupancy Level shows a very low negative correlation (−0.06), suggesting that the number of occupants has a negligible direct impact on EUI. The Window to Wall Ratio shows no correlation (0.00) with EUI, indicating that in this dataset, the proportion of windows to wall space does not significantly impact energy consumption.

Lastly, Lighting Density and Heating Setback Temperature show relatively lower correlations (0.24 and 0.19, respectively), suggesting a modest influence on EUI. These factors, while important, may not be as impactful as HVAC efficiency or insulation quality. In summary, this analysis reveals that HVAC efficiency, insulation, and domestic hot water usage are key drivers of energy use in Irish residential buildings.

## 4 Experimental Design, Materials and Methods

The primary motivation behind Irelandʼs residential building stock dataset is to employ a data-driven approach for assessing energy performance and predicting it on an urban scale [1]. The dataset generation process contains initial data requirement, building archetypes development, and parametric simulation.

### 4.1 Initial data requirement

In the experimental design, the first step is the gathering comprehensive urban buildings data is a challenging task, mainly due to the limited availability of specific building details. This initial data requirement process involves gathering raw building data from multiple sources to create an extensive database of urban building inventories. This data includes anonymous secondary data, such as building energy performance certificates, building census datasets, and weather information. These secondary data sources collectively contribute to generating a complete building stock dataset, making efficient use of available resources through parametric simulations. In the context of Ireland, data on residential buildings is largely derived from Energy Performance Certificates (EPCs), known locally as Building Energy Rating (BER) certificates, managed by the Sustainable Energy Authority of Ireland (SEAI). The dataset originating from EPCs is comprehensive, covering more than 200 attributes of buildings such as their construction, heating solutions, usage estimates, carbon dioxide emissions, and both estimated actual and theoretical energy usage. However, gap in Irish EPC data primarily due to the lack of dynamic elements and the challenge of incorporating projections of future conditions. Additionally, EPC data currently encompasses only about half of Dublin City's residential buildings. To address these challenge, this study aims to generate synthetic data that will encompass a comprehensive range of building characteristics, including those not yet known, to facilitate future analyses and machine learning modeling Similarly, the Central Statistics Office (CSO) of Ireland carries out a national census every four years, gathering data that includes information on the housing stock, thereby offering insights into the distribution of buildings across various locales. Consequently, the census provides information on the number of buildings in different geographic areas. Furthermore, weather data for Dublin is obtained from the standard EnergyPlus dataset, featuring historical records and projected weather patterns for 2030, provided by Meteonorm. This information is crucial for evaluating the influence of climate conditions on the effectiveness of building retrofitting strategies under different climate change scenarios.

### 4.2 Building archetypes development

**Fig. 2:**
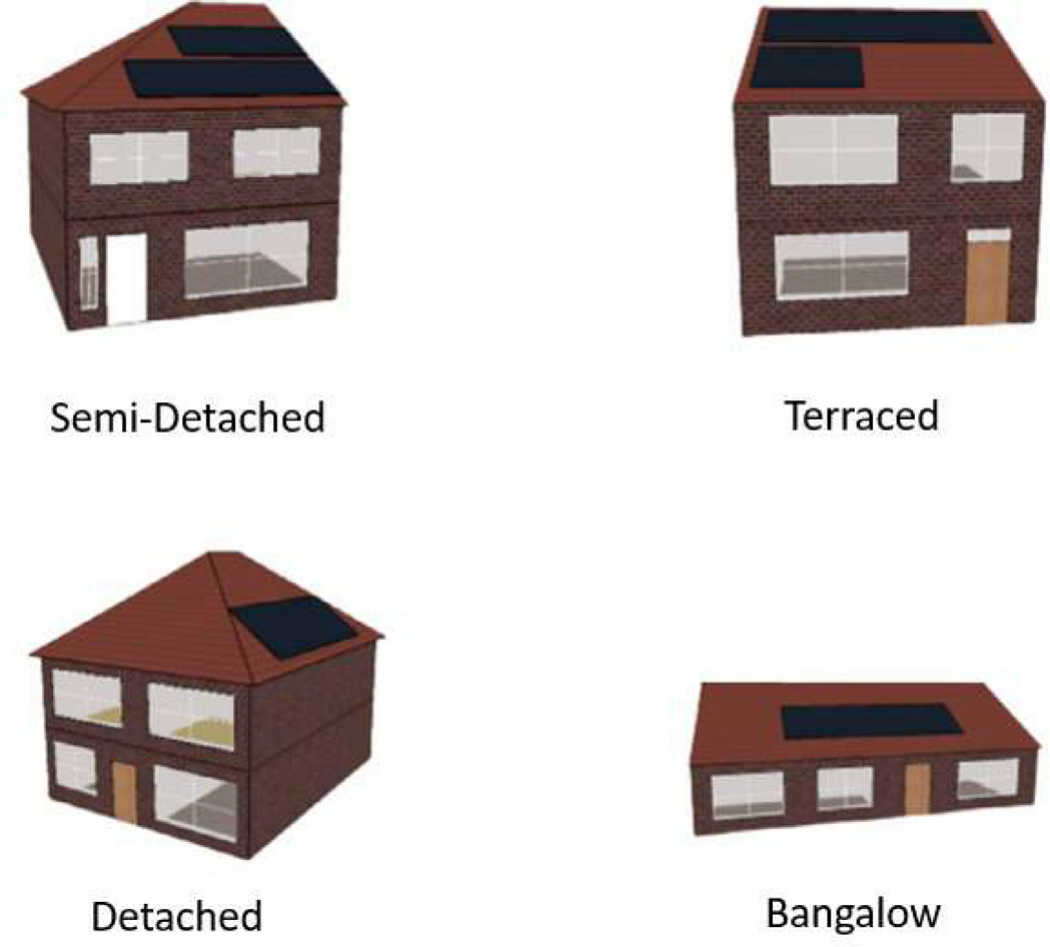
The geometric model of Irish residential building archetypes for energy parametric simulation for Dublin City [1].

In this study, the Irish residential sector is represented through four main building archetypes: terraced houses, detached houses, semi-detached houses, and bungalows (Fig. 2). Each building archetype serves as a benchmark model for parametric simulation analysis and aiding in the development of a synthetic representation of the building stock. Modeling these archetypes requires the collection of both geometric and non-geometric information.

The first phase entails the identification of these critical parameters from the current building stock in Dublin. Geometric information is gathered for different building types across Ireland, adhering to the standards prescribed by Irish building regulations. Non-geometric aspects are ascertained through the examination of existing databases on building energy efficiency and through comprehensive reviews of existing articles. For instance, data from the Irish Energy Performance Certificate (EPC) is invaluable, providing essential physics data for buildings, like the U-values for walls, roofs, floors, and windows, and their respective ranges. Through previous studies, other significant non-geometric elements that influence the energy efficiency of the Irish building stock have been pinpointed.

The compiled datasets encompass geometric details such as the total floor area, roof area, the ratio of window area to wall area, the number of floors, the number of distinct zones within a building, and its orientation. For example, the total floor area for terraced houses is recorded at 91.66 square meters, whereas detached houses exhibit a larger floor area of 130.81 square meters. These parameters are crucial for providing a deeper understanding of the energy efficiency and distinct characteristics of various types of buildings, enabling precise simulations and analyses of energy performance specific to the Irish context.

### 4.3 Parametric simulation

In the parametric simulation step, parametric selection is of utmost importance for creating synthetic datasets using building archetype. The accuracy of energy models for buildings depends significantly on the selection of both input and output variables, which must cover the range of variations essential for synthesizing data. This study incorporates 19 input variables to model archetypes of Irish residential buildings, inspired by prior research in the area [2]. However, certain advanced features were not addressed in these prior studies. As a result, we have integrated several additional parameters, including those related to HVAC systems, to conduct a comprehensive analysis encompassing HVAC (Heating, Ventilation, and Air Conditioning) systems, primary heating factors, and renewable parameters (Table 4).

**Table 4:** Parameters needed for parametric simulation of archetype.

Input parameters	Minimum	Maximum
Building Type	Semi Detached, Detached, House, Terrace, Bungalow	
Location	Dublin	
Weather	Historical, 2030	
Wall U-value	0.09	2.4
Window U-value	0.73	5.75
Floor U-value	0.15	1.6
Roof U-value	0.07	2.28
Door U-value	0.81	5.7
Orientation	0	315
Equipment Density	1	21
HVAC Efficiency/COP	0.3	4.5
Domestic Hot Water	0.5	3.5
Air Change Per Hour	0.35	3
Lighting Density	1	9
Occupancy	1	6
Heating Setpoint	18	23
Heating Setback	10	14
Window-to-Wall Ratio	0	70
Renewables	Yes/No	

Moreover, the proposed approach simplifies the model by using Design-Builder templates to represent construction specifics, thereby reducing the complexity of interrelated variables. For example, the physical attributes of building materials—like their thickness, conductivity, density, and heat capacity are represented through U-values in existing templates. This method effectively narrows down the input variables needed for UBEM, enhancing computational efficiency by omitting variables that are dependent on each other.

Energy Use Intensity (EUI) is a principal metric in this analysis, measuring a building's annual primary energy usage per square meter of total floor space, expressed in kWh/(m^2^·year). The Energy Performance Certificate (EPC) data from Ireland offers deep insights into building energy efficiency, classifying buildings on an A1 to G scale based on their EUI values. An A1 rating indicates the highest energy efficiency, characterized by lower energy use and CO_2_ emissions, while a G rating denotes the lowest efficiency. The dataset encompasses data on heating, lighting, devices, solar photovoltaic systems, and water heating, providing a holistic view for analysis.

An A1-rated building represents the pinnacle of energy efficiency, often associated with lower energy consumption and reduced CO_2_ emissions. In contrast, a G-rated building signifies the lowest level of energy efficiency. Furthermore, this dataset contains information pertaining to heating, lighting, equipment, photovoltaic systems, and hot water, making it a comprehensive source of data for analysis. In this methodology, jEPlus is employed as the parametric tool for physics-based parametric simulation. jEPlus uses EnergyPlus for thermal simulation and integrates DesignBuilder construction templates to incorporate diverse parameter values. EnergyPlus is a widely-used building energy simulation software, serving as the core thermal simulation engine within jEPlus. It accurately models the thermal behavior of buildings by considering factors such as heating, cooling, lighting, and more. EnergyPlus requires climate data, geometric and non-geometric input data for simulations. Weather data is crucial as it determines external conditions throughout the year. The chosen Dublin City EnergyPlus Weather (EPW) file provides detailed weather data for Dublin, Ireland. Information about the building's geometry is shown in (Fig. 2). Similarly, consider 19 input parameters for the simulation of each building archetype. The validation process involves comparing parametric simulation results with real-world data or benchmark values. The developed archetypes are validated in existing studies using the current Irish EPC software for building performance analysis.

The challenge of simulating data across a wide array of parameter combinations is formidable due to their complexity. Sampling methods such as Simple Random Sampling (SRS) and Latin Hypercube Sampling (LHS) are commonly used for synthetic data generation. SRS is a basic method where each sample is chosen randomly and independently from the population. On the other hand, LHS is a more sophisticated approach, striving for a more evenly distributed sample spread across the data's entire range. LHS is designed to maintain a balanced combination of parameter values, facilitating a more thorough design space exploration. This study uses the LHS method to generate a sample of 1 million buildings, aiming to build a robust machine-learning model. This approach ensures comprehensive coverage of the energy rating data for Irish buildings in the resulting distribution.

## Limitations

The data required to generate a synthetic dataset of 1 million buildings, including building geometry, non-geometric data, census information, and weather data, originate from various sources and come in different formats. This leads to data inconsistencies. Consequently, these inconsistencies and the absence of standardized urban-scale data present a significant and ongoing barrier. However, it is essential to note that the accuracy and implementation of the model depend on the quality and availability of input data, which may vary in different contexts and countries. Moreover, developing synthetic data for various building archetypes in different contexts might require additional computational time. The primary limitation of this study arises from its reliance on pre-defined geometric parameters to construct different building archetypes. While this approach allows for a comprehensive exploration of various design possibilities, it overlooks the practical constraints imposed by non-geometric factors. In real-world scenarios, buildings are often shaped by a multitude of non-geometric parameters such as construction materials, occupancy behavior, and economic considerations. These factors significantly influence the final architectural form and its energy efficiency. Furthermore, the value and further use of the dataset generated in this study must be examined in light of this limitation. For practitioners, policymakers, and researchers interested in energy-efficient building designs, the utility of this dataset hinges on its ability to reflect realistic and commonly encountered building configurations. Future studies could focus on integrating a more balanced approach that considers both geometric and non-geometric parameters, thereby ensuring that the resulting dataset is diverse and reflective of typical building types and refurbishment practices observed in various regions and periods.

## Ethics statement

The authors have read and follow the ethical requirements for publication in Data in Brief and confirming that the current work does not involve human subjects, animal experiments, or any data collected from social media platforms.

## CRediT authorship contribution statement

**Usman Ali:** Conceptualization, Methodology, Writing – original draft. **Sobia Bano:** Writing – review & editing. **Mohammad Haris Shamsi:** Writing – review & editing. **Divyanshu Sood:** Writing – review & editing. **Cathal Hoare:** Writing – review & editing. **Wangda Zuo:** Writing – review & editing. **Neil Hewitt:** Writing – review & editing. **James O'Donnell:** Supervision, Conceptualization, Writing – review & editing.

## Data Availability

Urban Building Energy Stock Datasets (Original data) (Mendeley Data).
